# Anti-Tumor Effect of Parasitic Protozoans

**DOI:** 10.3390/bioengineering9080395

**Published:** 2022-08-16

**Authors:** Haojie Ding, Songrui Wu, Zi Jin, Bin Zheng, Yuan Hu, Ke He, Shaohong Lu, Xunhui Zhuo

**Affiliations:** 1 School of Basic Medical Sciences and Forensic Medicine, Hangzhou Medical College, Hangzhou 310059, China; 2 Key Laboratory of Parasite and Vector Biology, National Health Commission of People’s Republic of China, National Institute of Parasitic Diseases, Chinese Center for Disease Control and Prevention (Chinese Center for Tropical Diseases Research), WHO Collaborating Center for Tropical Diseases, Shanghai 200025, China; 3 College of Animal Science and Technology, College of Veterinary Medicine, Zhejiang Agriculture and Forestry University, Hangzhou 311300, China

**Keywords:** anti-tumor, protozoon, biotherapy, immune response, tumor microenvironment

## Abstract

The immune system may aberrantly silence when against “altered self”, which consequently may develop into malignancies. With the development of tumor immunology and molecular biology, the deepened understanding of the relationship between parasites and tumors shifts the attitude towards parasitic pathogens from elimination to utilization. In recent years, the antitumor impact implemented by protozoan parasites and the derived products has been confirmed. The immune system is activated and enhanced by some protozoan parasites, thereby inhibiting tumor growth, angiogenesis, and metastasis in many animal models. In this work, we reviewed the available information on the antitumor effect of parasitic infection or induced by parasitic antigen, as well as the involved immune mechanisms that modulate cancer progression. Despite the fact that clinical trials of the protozoan parasites against tumors are limited and the specific mechanisms of the effect on tumors are not totally clear, the use of genetically modified protozoan parasites and derived molecules combined with chemotherapy could be an important element for promoting antitumor treatment in the future.

## 1. Introduction

Cancer is a growing global disease with no borders. It is regarded as one of the leading causes of mortality in humans, with an estimated 19.3 million new cancer cases and almost 10.0 million cancer deaths occurring in 2020, according to Global Cancer Statistics [1]. The development of efficient therapeutic strategies is important. In addition to traditional cancer treatments including surgery, radiotherapy, and chemotherapy, more recently, cancer biotherapies such as cancer vaccines, monoclonal antibodies, gene therapy drugs, and immunomodulators have been used clinically and studied in clinical trials. By killing cancer cells or inhibiting their growth, the mechanism of biotherapy mainly induces defensive immune responses against tumors in multiple targets and directions, in contrast to traditional treatment [2]. In recent years, some parasites, especially intracellular protozoan parasites, have been considered effectors to induce anti-pathogen and anti-tumor immune responses so as to overcome tumor escape and active tumor surveillance system. The persistent antigen release caused by the chronic infection of protozoan parasites leads to a long-term specific immune response, which may have broader advantages for promoting cancer treatments. This review outlined the mechanism of the anti-tumor effect of parasitic protozoon, aiming to provide novel strategies for the clinical treatment of tumors.

## 2. Tumor Therapy with the Injection of Parasite

**Figure 1:**
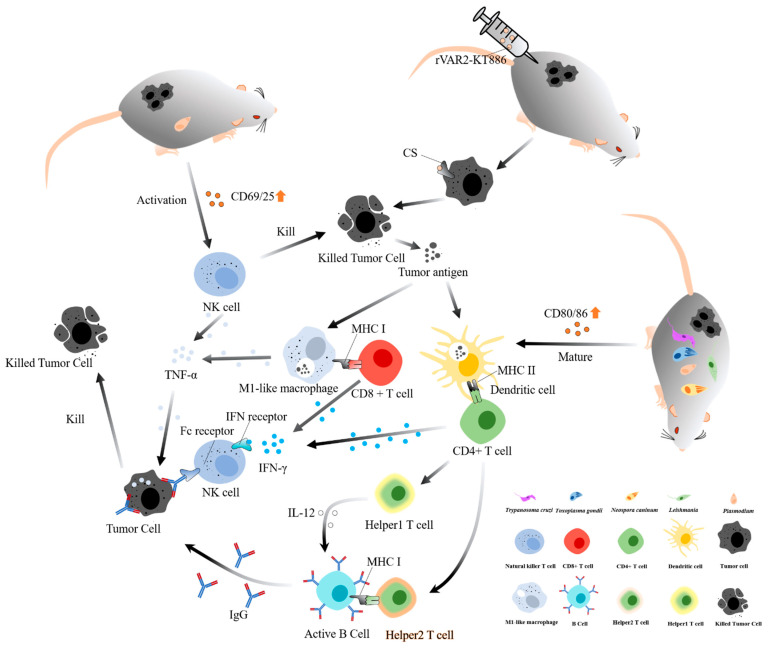
The landscape of the host’s immune system activated by parasites against the tumor. There are three ways that parasites could stimulate the host’s immune system to eliminate tumors. (1) Plasmodium could boost the level of CD69/25 and promote the natural killer T cell activation to secrete TNF-α; (2) RVAR2-KT886, a targeted drug based Plasmodium rVAR2, could recognize the CS in the surface of tumor cells and then kill it; (3) *Toxoplasma gondii*, *Leishmania*, *Trypanosoma cruzi*, *Neospora caninum* and *Plasmodium* could boost the level of CD80/86 and promote the dendritic cell maturity, and the mature dendritic cell actives CD4+ T cell to be Help1 T cell or Help2 T cell through MHC II. Help1 T cell would kill tumor through IL-12/IFN-gamma Axis; Help2 T cell would stimulate the B cell to produce specific IgG against tumor cell. Abbreviations: TNF-α: tumor necrosis factor alpha; IFN-γ: Interferon Gamma; IL-12: Interleukin 12; MHC I: major histocompatibility complex class I; MHC II: major histocompatibility complex class II; CS: chondroitin sulfate.

Recently, the use of non-pathogenic live protozoan parasites as anticancer therapeutic approaches has drawn attention worldwide, of note is the long history of antitumor effect by *Trypanosoma cruzi.* At the very beginning in 1946, the work from the former Soviet Union reported that cancer is rare in patients infected with *T. cruzi* once before [3], which opened possibilities for research on cancer biotherapy. Later, researchers developed an anticancer experiment in which *T. cruzi* extracts were directly inoculated in peritumoral areas over different tumors, and all of the results showed a reduction in tumor size [4]. However, the work was hampered by controversial results and the complicated international political situation, and so the molecular basis of this phenomenon has remained elusive [5]. Moreover, several live-attenuated protozoan parasites including *Leishmania infantum* and *L. tropica* [6], *Neospora caninum* [7], and *Toxoplasma gondii* [8] have all lately been employed as antitumor biological agents via intratumoral injection, all of which decreased tumor development and local inflammation (Figure 1). The injection of live *N. caninum* tachyzoites either in or remotely from the tumor, successfully treated murine thymoma EG7 by strongly activating the natural killer cell- (NK cell-) and CD8-T cell-dependent protective antitumor response associated with interferon (IFN)-γ secretion in the tumor microenvironment, resulting in the lysis of the cancer cell [7]. Researchers from Ege University Medical School first reported that intratumoral administration of attenuated *Leishmania* strains in 4T1 breast cancer-bearing mice promoted M1 dominant activation of macrophages in spleen and tumor tissues with induction of proinflammatory cytokines that helps the generation of protective Th1 responses [6,9]. It is a pity that the survival rate and other antitumor effects were not recorded. Further studies on the molecular basis and the effect on different tumors needs to be carried out to deepen the understanding and application of antitumor biotherapy with *Leishmania* strains.

The parasite *T. gondii*, on the other hand, attracts more attention. The characteristic of *T. gondii* that can infect nearly all types of cells and modify the immune response of the host opens up a wide range of clinical possibilities for *T. gondii* as an oncolytic protozoan in human medicine [10]. Considering that the toxoplasma virulence is cell type-independent, researchers in 1985 performed an intralesional injection with 10^7^ formalin-fixed *Toxoplasma* tachyzoites one day after the syngeneic Lewis lung carcinoma cell inoculation and discovered some mice (2/6) completely rejected the growth of the implanted tumor [11]. It is conceivable that live organisms could elicit a much more robust antitumor effect than dead ones. Thus, what followed was gamma radiation-attenuated Toxoplasma [12], and gene-edited-attenuated Toxoplasma such as *ΔCPS*, *ΔOMPDCΔUP*, *ΔGRA17* strains, and *Δldh1*-*Δldh2* strains used for tumor biotherapy, which all presented the ability to repress the growth of established tumors and to help the inhibition of lethal tumor development in the mice [8,13,14]. Overall, these protozoan parasites are proven to have effective antitumor effects, while the precise targets of tumor cells need further investigation. Referred to as the chimeric antigen receptor (CAR)-T method, whether the combination of protozoan parasites and tumor-specific antigens would promote the target invasion and antitumor effects requires further research data.

## 3. Antitumor Effect of Parasitic Products

Some parasitic products have been demonstrated to have specific antitumor effects. It is well known that the levels of aberrant chondroitin sulfate proteoglycans (CSPGs), a protein family that displays one or multiple chondroitin sulfate (CS) side chains, are upregulated in many cancers but the variability of this protein within different tumor tissues is striking, due to the great diversity of structure cores assembled by numerous enzymes regulated, based on tissue and cell type [15,16]. The CS side chains or the protein core of CSPGs can bind extracellular matrix components or a growth factor receptor complex to transmit pro-oncogenic signaling through which the proteins are implicated in cancers. The identification and targeting of CS in cancers remained a technical challenge until the specific CS structure, termed oncofetal CS (ofCS) was found to share a high affinity among cancer cells [17]. A refined malaria protein called rVAR2 was discovered to bind with the distinct ofCS from a variety of different cancer cell lines [18], with remarkably high specificity and affinity (KD ~15 nM) [19]. This discovery was further supported by the observation that rVAR2 intravenously injected, adhered to the tumors in in vivo xenograft animal models [20]. The high efficiency targeted tumor characteristic of rVAR2 makes it a potentially ideal carrier for anti-cancer drug delivery. Indeed, it has been proven that a hemiasterlin analog (KT886) conjugated rVAR2 (VDC886), formed as the complex which carried an average of three toxins per rVAR2 molecule, effectively kill a total of 33 cancer cell lines in vitro with inhibitory concentration 50 (IC50) values ranging from 0.2 pM to 30 nM [18]. Remarkably, the VDC886 treatment significantly slowed the growth of both non-Hodgkin’s lymphoma (Karpas299) and prostate cancer (PC-3) in mouse models, indicating a potential role in clinic therapy.

Unlike malaria VAR2, rhoptry and dense granule proteins secreted by *T. gondii* play advantageous roles in regulating the growth of tumors. Two key organelles, the apical rhoptry [21] and dense granule [22] are essential for host invasion and immune escape. Through a reverse genetic approach to target complete gene deletions, particular proteins including rhoptry protein 5 (ROP5), ROP17, ROP18, ROP35, and ROP38, the dense granule protein 2 (GRA2), GRA12, and GRA24 are demonstrated to effectively activate antitumor immune responses involving CD4+ and CD8+ T cells and the interleukin-12 (IL-12)/IFN-γ T_H_1 axis, while deletion of GRA3, GRA15, GRA16, ROP16, or ROP21 did not affect the antitumor activity [23]. Coincidentally, the secreted GRA15 was confirmed to localize to the endoplasmic reticulum and to activate innate immune and stimulator of interferon genes (STING) responses by promoting polyubiquitination at Lys-337 and oligomerization in a tumor necrosis factor (TNF) receptor-associated factor (TRAF) protein-dependent manner [24]. In addition, the GRA16 displayed the ability to break the chemoresistance of irinotecan by inhibiting nuclear factor kappa B (NF-κB) via a PP2A-B55/AKT/NF-κB p65 pathway against non-small-cell lung carcinoma (NSCLC) [25]. Recently, the fact that the small intestine (SI) exhibited low tumorigenesis or metastatic growth from distant tumors attracted attention. Concomitantly, a protein named *Eimeria* antigen (EA) that acts as a robust stimulator to promote IL-12 releasing from dendritic cells (DC) and to upregulating inflammatory modulators (Monocyte chemoattractant protein-1 (MCP-1), IL-6, IFN-γ, and TNF-α) for the defending of sarcoma tumor in mice was discovered [26]. Perhaps the vast immunologic compartment induced by EA in SI also provides a potent tumor immunosurveillance. These findings collectively point to a deeper understanding of fundamental mechanisms of host cell pathways manipulated by these secreted proteins, which could provide novel targets for developing effective therapies against aggressive solid tumors.

Another well-known anti-tumor protein is the calreticulin from *T. cruzi* (TcCRT), which is secreted into the extracellular milieu to induce immune modulations [27,28]. TcCRT consists of an N-terminal vasostatin-like domain (aa 20-193) that can directly bind with endothelial cells through a scavenger-like receptor and acts as a potent angiogenesis inhibitor [29,30]. As we all know, angiogenesis-focused therapy is frequently utilized in conjunction with other treatments for a variety of tumor types [31,32]. Most likely, by directly interacting with endothelial cells, the N-terminal vasostatin-like domain inhibits vascular endothelial growth factor (VEGF)-induced cell proliferation and induces cell apoptosis [33]. TcCRT makes use of its anti-tumor properties in this manner.

## 4. Activating the Cellular Immune System

Antitumor immunity including innate and adaptive immune responses, mainly activated depending on the close interaction with several elements, has the most important role in tumor control [34]. However, tumor cells often evolve many mechanisms to escape immune surveillance and elimination [35]. Therefore, disrupting these mechanisms and promoting tumor cell recognition has the potential to effectively boost anticancer immunity. In some way, some protozoan parasites and their products may facilitate targeting tumor cells and induce positive immune responses, yielding long-term benefits of immunity against tumors, such as immune cells and potent cytokines.

### 4.1. Immune Cells

Cells of innate immunity and adaptive immunity, including NK cells, DC, Innate lymphoid cells (ILC), cytokine-induced killer cells (LAK), specific T lymphocytes, etc., play important roles in tumor immunotherapy by controlling tumor growth and killing tumor cells.

Acting as toxic immune cells, activated NK cells managed by a suite of activating, co-stimulatory and inhibitory receptors [36], can directly kill tumor cells, especially those that lack major histocompatibility complex (MHC; also known as human leukocyte antigen (HLA)) class I [37], such as tumor cells from metastatic and blood tumors, and indirectly improve the response of antibodies and T cells [38]. By the stimulatory infection of *Plasmodium*, NK cells can be activated to kill some lung cancer cells which in return can release tumor antigens resulting in activation of the systemic response of tumor antigen-specific T cells in peripheral blood, spleen, and lymph nodes, as the consequence of additional cancer cell death [39]. Possibly, the toxic activation of NK cells via IFN-γ expression is induced by the infection of Plasmodium through the up-regulated expression of CD69 and CD25 [40]. As reported by Chen, et al. [41], three clinical trials based on *Plasmodium* immunotherapy against advanced cancers have been approved and are underway with clinical safety guaranteed. In addition, the infection of *N. caninum* [7], *Leishmania amazonensis* [42], and *T. gondii* [43] increased the levels of NK cells mainly through an IFN-γ-dependent pathway. Moreover, toxoplasma infection can induce the conversion of NK cells into ILC1-like cells which are Eomes-dependent and the changes appear permanent [44], which may help defend against tumors. However, the molecular mechanism of how these protozoans activate NK cells is not well studied. While the role of NK cells in controlling tumor growth is well established, the function of newly discovered ILCs in defending tumors remains poorly understood. Nevertheless, their ability to produce large amounts of cytokines [45,46] indicates that ILCs may contribute to tumor-associated inflammation, and the interaction between protozoan parasites and ILCs may shed light on the development of new antitumor therapies. Genetic modification with superior activation or decreased inhibitory signals in NK cells enhances their tumor cell killing ability [47]. It is conceivable when using the specific parasitic molecular, such as VAR2 form *Plasmodium*, as the chimeric antigen receptor (CAR) in NK cells, the most effective CARs used to redirect T cells also work well for NK cells [48,49].

Undoubtedly, T cells play crucial roles against tumors [50]. Activation of naïve T lymphocytes requires T cell receptor (TCR) signaling, costimulatory signaling, and cytokine support [51,52]. A neoantigen peptide, uniquely encoded by mutated DNA of tumor cells, has distinct epitopes from those of normal cells and will be processed and displayed on the surface of tumor cells and antigen-presenting cells (APC) as the form of neoantigen peptide-major histocompatibility complexes (pMHC) [53,54]. During T cell activation, the expressed membrane proteins CD4 on T helper cells and CD8 on cytotoxic T lymphocytes, bind to MHC class II (MHC-II) or MHC-I molecules, respectively [55]. However, recent evidence revealed that the multifaceted suppressive signals resulted in an exhausted phenotype or dysfunctional state of T cells [56,57]. Hence, effective therapeutic immunity against tumors can be stimulated by reversing tumor-associated immunosuppression. So far, a few studies have indicated that this goal can be addressed using the infection of protozoans, which may reverse T cell suppression and activation by providing exogenous antigens. Mice immunized with live-attenuated *Leishmania* parasites presented a higher percentage of CD4+ and CD8+ T cells, indicating a robust cellular response was generated [58,59], consequently these T cells infiltrated the 4T1 breast cancer resulting in the decrease of tumor volume and prolonged the survival period of mice [6]. Similarly, vaccination with *T. cruzi* significantly inhibited the growth of breast and colon tumors through the activation of CD4+ and CD8+ T cells and the increase of macrophages and dendritic cells. A diverse cell-mediated (CD4+ and CD8+ T cells) immunity lasted more than 200 days in mice that were triggered by an attenuated *T. gondii* against pancreatic cancer recurrences [60].

Although DC cannot directly kill tumor cells [61], they are the cornerstone of the anti-tumor immune response due to the ability to activate T cells by extracting and transporting specific tumor antigens making them the foundation of the anti-tumor immune response [62]. However, it is usually insufficient for DC maturation due to the suppressive mechanisms within tumors [63], including suppressing the expression of MHC or costimulatory molecules [64]. Some protozoan parasites may help promote the mutation of DCs, and this notion is supported by studies demonstrating that ligand molecules from parasites bind with Toll-like receptors (TLRs) to stimulate DC activation, such as glycosylphosphatidylinositol (GPI) from *Leishmania major* [65], *T. cruzi* [66], *P. falciparum* [67], and *T. gondii* [68], and Profilin-like protein from *T. gondii* [69]. In mice implanted with murine Lewis lung cancer (LLC) cells, malaria parasite infection promotes the maturation of DCs through up-regulating the expression of CD80 and CD86 resulting in the activation of T cells [39]. Recently, Lantier, et al. [7] found that unlike dead parasites or soluble tachyzoites antigens, live *N. caninum* tachyzoites are able to activate murine or human DCs to secret proinflammatory cytokines, which convincingly suggests that the use of live tachyzoites is a necessary condition for immunotherapeutic treatment. In fact, four days after the injection of live tachyzoites, they observed recruitment of DCs, along with a high increase of IFN-γ and IL-12, indicating a measurable and systemic immune response against EG7 thymoma in mice. Coincidentally, Baird, et al. [14] found that CD11c^+^ DC antigen-presenting cells from the ovarian carcinoma microenvironment invaded by the infection of toxoplasma cps strain, strongly upregulate the costimulatory molecules CD80 and CD86, which regained the ability to cross-present antigen to prime tumor antigen-specific CD8+ T cell responses.

### 4.2. Cytokines

Cytokines are major regulators of innate and adaptive immunity, and some play critical roles in tumor cells [70]. Among these molecules, IFN-γ can induce tumor cell cycle arrest and establish tumor cell dormancy [71], and IL-12 can promote Th1 antitumor immune response which may be secreted by the stimulation of IFN-γ and in turn triggers the re-activation of the IFN-γ production cycle [72], while IL-10 and IL-13 can inhibit Th1 cells from secreting IFN-γ [73]. The highly expressed IL-6 can promote inflammation and tumor cell immunosuppression [74], and possibly aggravate tumor cell growth and metastasis by IL-6/Signal Transducer And Activator Of Transcription 3 (STAT3) mediated inhibition of DC and lead to the dysfunction of the immune system [75]. Generally speaking, cytokines dominated by IL-6 are beneficial for tumor proliferation and metastasis, while these inflammation factors mediated by IFN-γ have an anti-tumor effect on blocking tumor progression [76]. For instance, exposure to Toxoplasma tachyzoites, in addition to activating immune cells as mentioned above, can induce the antitumor effect in mice models by increasing the expression levels of IFN-γ [8,13,23,43,77], IL-12 [14,23,43,78], and TNF-α [8,13].

## 5. Activating the Humoral Immunity System

A long-term, effective anti-tumor immune response is essential for the treatment of tumors, and during this period, some tumor-associated antigens (TAA), which are at very low levels in normal cells, will be recognized and prime the humoral immunity [79]. The auto-tolerance prevents the direct recognition against self-antigen, but the infection of protozoan may help induce the anti-tumor humoral immunity followed by the releasing of antibodies which can affect the biology of the tumor by blocking certain receptors on the surface of tumor cells [80]. Infection of toxoplasma ME49 strains significantly increase the levels of IgG1 and IgG2a in LLC-bearing mice [77], and, live *L. tarentolae* carrying E7 protein induced significant levels of IgG2a against HPV-associated tumors [81]. It would be more convincing if the tumor-specific IgG was detected. The similarity between surface antigens of *T. cruzi* and Ehrlich’s adenocarcinoma cells was confirmed by the cross-reaction of indirect immunofluorescence [82]. Later, the sera from *T. cruzi* lysate-vaccinated mice significantly reduced the tumor size of Ehrlich’s adenocarcinoma. This could imply that the specific immune profile generated by antigens of *T. cruzi* has a positive effect on the growth of a tumor, at least regarding this type of neoplasm.

## 6. Suppressing the Angiogenesis and Tumor Metastasis

Angiogenesis, the process of recruiting new blood vessels, is an essential component of tumor metastasis to acquire adequate nutrients and oxygen to facilitate the spread and growth of tumor cells. Among the proangiogenic molecules, the acidic and basic fibroblast growth factors (aFGF and bFGF), the platelet-derived endothelial cell growth factor (PD-ECGF), and especially the VEGF are well studied [83]. Attenuated *T. gondii* ME49 remarkably suppressed the levels of angiogenic factors (VEGF, integrin, matrix metallopeptidase (MMP) 2, and MMP9) in an Ehrlich ascites carcinoma cancer model, which in turn inhibited neoplastic growth [12]. It is interesting to note that protozoan parasites suppress neovascularization through quite different pathways. For instance, the infection of Plasmodium exhibited an inhibitory effect on hepatocellular carcinoma angiogenesis, unlike the effect of *T. gondii* mentioned above, further detection showed no significant decrease in VEGF levels [84]. According to many reports, VEGF is known as a major proangiogenic molecular released by tumor-associated macrophages (TAMs) and is correlated with many human cancers [85]. Later, they discovered the diminished angiogenic responses in *Plasmodium*-infected tumor-bearing mice probably by decreasing the expression of angiogenesis-related enzyme MMP9 via the insulin-like growth factor (IGF) axis through the PI3-K and MAPK signaling pathways [41,84]. In addition, the parasite-derived antigens have an effort on inhibiting vascularization in tumors as well. It has been reported that *T. cruzi* CRT or more precisely, its N-terminus, directly interacts with endothelial cells through VEGF and inhibits angiogenesis in breast tumors in vitro, ex vivo, and in vivo [86,87]. In the treatment of mice with *T. gondii* lysate antigen (TLA) in the murine sarcoma-180 tumors model, both the decreased expression of CD31 (an angiogenesis marker in the tumor tissue) and the reduction in tumor size were observed [88].

## 7. Improving the Microenvironment

**Figure 2:**
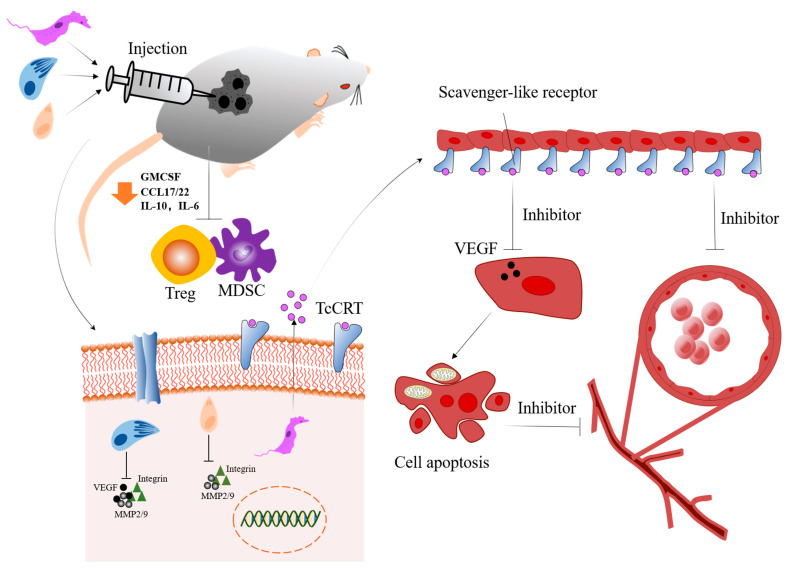
Protozoans modulate the tumor immune microenvironment and inhibit angiogenesis. *Leishmania*, *Neospora caninum* and *Toxoplasma gondii* could downregulate some cytokines (GMCSF, CCL17/22, IL-10 or IL-6) to inhibit Treg and MDSC; besides, these protozoans also could inhibit angiogenesis through suppressing the level of angiogenic factor (VEGF, MMP2/9 or integrin). Among these, the scavenger-like receptor could recognize TcCRT secreted by *Trypanosoma cruzi*, and it could inhibit VEGF to induce cell apoptosis. Abbreviations: MDSC: myeloid-derived immunosuppressive cell; Treg: regulatory T cells; GMCSF: Granulocyte-Macrophage Colony-Stimulating Factor human; CCL17/22: C-C class chemokines 17/22; IL-10: Interleukin 10; IL-6: Interleukin 6; VEGF: vascular endothelial growth factor; MMP2/9: matrix metallopeptidase 2/9; TcCRT: *Trypanosoma cruzi* calreticulin.

Cancer immunotherapy based on immune checkpoint blockade has shown its value in clinical treatments, but unfortunately, only a small number of tumor-bearing patients benefit from the therapy. The failure is likely due to the low immunogenicity, high numbers of immunosuppressive cells, and insufficient T cell infiltration in the tumor microenvironment (TME) [89]. Myeloid-derived immunosuppressive cells (MDSCs), a group of immature myeloid cells at different development stages, can suppress anti-cancer immune responses by inhibiting the antitumor effects of CD8+ T cells [90]. Regulator T cells (Tregs) are a subset of immunosuppressive T cells that are recruited into the tumor microenvironment by interacting with various tumor-secreted chemokines [91]. MDSCs and Tregs are believed to play a major role in the suppression of antitumor immune responses [92,93]. There are some reports that *Plasmodium* and *T. gondii* can reduce the number of immunosuppressive cells (Figure 2). For instance, in the *Plasmodium yoelli* infected tumor-bearing mice, several MDSC- and Treg- recruiting cytokines and growth factors were downregulated in the tumor microenvironment. Plasmodium infection could inhibit tumor secretion of Granulocyte-Macrophage Colony-Stimulating Factor human (GMCSF), IL-10, IL-6, C-C class chemokines (CCL)-17, and CCL-22 through the release of exosome-like vesicles [94]. The CCL17/22-C-C chemokine receptor4 (CCR4) pathway is the major pathway in the recruitment of Tregs into the tumor microenvironment [94]. Besides, attenuated *T. gondii* infections do not inhibit the recruitment of Treg cells. Foxp3 expressing cells do not change in the Pancreatic ductal adenocarcinoma (PDAC) tumor microenvironment [95]. Moreover, non-replicating *Toxoplasma* uracil auxotrophs (NATUA) and Carbomyl phosphate synthetase (cps) gene knockout strains show that these parasites can enhance the T cell infiltration and improve the tumor immunogenicity of the tumor. For instance, combination therapy of attenuated *T. gondii* NRTUA with anti-programmed death-1 (PD-1) led to elevation of CD8+ T cell infiltration mediated by dendritic cell-secreted IL-12 and to tumor-specific IFN-γ production in the PDAC tumor microenvironment [96]. CD8+ T cell infiltration in the TME will be reduced with the depletion of IL-12. Combination therapy of NRTUA with anti-PD-1 also significantly reduced tumor weight in PDAC solid tumor [96].

## 8. Conclusions and Future Directions

In this review, we have gathered information about antitumor effects induced by protozoan parasites, and the associations between infection with protozoan parasites and cancers are well-evidenced. Parasites and some derived products may exert beneficial antitumor responses which could serve as new strategies to treat and prevent these diseases. Perhaps these antineoplastic effects induced by the protozoan parasite reflect the evolution to protect itself as well as the integrity of the host. As mentioned in Table 1, these parasites, including *T gondii*, *Plasmodium*, *Leishmania*, *N. caninum*, *T. cruzi*, and *Eimeria*, and also the secreting proteins, have direct or indirect effects on cancer cells or on the tumor microenvironment, resulting in halting cancer growth or eliminating cancer in mice models. However, the mechanisms involved in cancer development regulation triggered by protozoans are diverse and not yet fully described, and even the related types of tumors are very limited. Even though protozoan parasites are unicellular eukaryotes with similar sharps, the host cell, life cycle, and virulence are distinct, thus the activated immune effects on carcinogenesis are not the same. Apart from the above factors, the variability of antitumor responses may count on the following factors: the type of cancer cell and its transformation stage or even the tissue type and location in which it is found, the inoculation method, the vitality of parasites, as well as the regulated immune responses generated by the host. Most protozoan parasites could elicit unspecific activation of immune cells, i.e., DC and NK through different signaling pathways, and induce antitumor cytokines, such as IFN-γ, IL-12, and TNF-α.

Nevertheless, these antitumor studies are mainly limited to animal models, there is no data evidence from clinical trials to support these results in human patients. The consideration of such parasite-dependent intervention focuses on the harm to the host and unexpected infection caused by injecting live parasites, which may hamper the results of clinical trials, and lead to a practical problem about how to avoid immune damage caused by parasitic infection when using the infection to fight tumors. Of note is the famous example that *T. cruzi* and its extracts which exhibited toxic effects over different tumors both in experimental animals and humans about 80 years ago. However, the complicated political and international situation interrupted this work. One possible solution to this concern is to identify parasite-derived products and associated molecules with toxic effects on cancer cells and on the microenvironment or that possess binding cancer cell features. Several in vitro and in vivo studies demonstrated that a malaria protein rVAR2 can directly bind with distinct ofCS from a wide range of different cancer cell lines, as confirmed by intravenous injection of rVAR2 in xenograft animal models. Furthermore, the calreticulin of *T. cruzi* can directly bind with endothelial cells and work as a potent angiogenesis inhibitor through a VEGF-dependent pathway. It is an impractical idea, at least now, to replace drug treatment with derived parasitic products given the fact that only limited clinical evidence was obtained based on these molecules, but the features of these products open the possibilities via acting as adjuvants or drug carriers to conduct comprehensive or combination therapies for improving the effectiveness of current drugs on cancers. In line with this idea, according to the preliminary in vitro analysis, a hemiasterlin analog (KT886) conjugated rVAR2 (VDC886) is demonstrated to effectively kill a total of 33 cancer cells, and in addition, the efficacy of VDC886 treatment is confirmed as effective by inhibiting the growth of karpas299 and PC-3 cancer in mouse models. Unlike the live parasite, the antitumor effect of VDC886 is mainly dependent on the drug KT886, as the cancers were effectively bound by rVAR2 in a concentration and CS-dependent manner rather than the involved signaling of HLA molecules. Therefore, it is essential to identify more parasitic derivative products and search for more effective drugs for potential use in antitumor treatments, as well as to illustrate their actions in regulating immune signaling.

Another major concern is the effectiveness of parasitic immunotherapy. To what level of targeting ability can clinical parasitic infection reach? Up to now, most antitumor studies of live attenuated protozoan parasites were focused on *Plasmodium* and *Toxoplasma* strains. As for *T. gondii*, crucial genes of the *de novo* pyrimidine synthesis pathways were genetically disrupted or knocked out so that the attenuated strains can normally invade host cells but lose the intracellular proliferation ability, thus eliminating the potential harm to normal host cells. In accordance with that mentioned above, the contingent risk or side effect can be outweighed compared with the fatal results of most malignant tumors out of the fact that these protozoan parasites are opportunistic pathogens, let alone the applied strains being genetically or gamma radiation-attenuated with limited pathogenicity. In addition, the immunotherapeutic attenuated *T. gondii* strains have been demonstrated in several mouse models by peritoneal or intratumoral injection by activating native and adaptive immune responses and affecting the tumor microenvironment. Thus, key research should be conducted for the clinical use of immunotherapeutic attenuated protozoan parasites in the next few years. It is worth noting that three clinical trials of *Plasmodium* immunotherapy for advanced lung cancer (NCT02786589), breast and liver cancers (NCT03474822), and advanced cancers (NCT03375983) have been approved and are ongoing in China. In recent years, cancer immunotherapy, especially the adaptive chimeric antigen receptor T (CAR-T) cell therapy, and immune checkpoint blockade therapy have been recognized as leading breakthroughs. Imagine if we combined CAR-T and checkpoint blockade therapy with attenuated protozoan parasites? For example, *Plasmodium* and *Toxoplasma* strains expressed with particular ‘chimeric antigen receptor’, i.e., PD-1 or CTLA-4, so as to increase the targeting ability of cancer cells. In this way, the ‘cold’ tumors can be turned into ‘hot’ ones by the infection of parasites, and the inactivated ‘sleeping’ immune cells can be wakened by checkpoint inhibitors and able to recognize and attack cancer cells. The so-called CAR-*Plasmodium* or CAR-*Toxoplasma* may be an impractical crazy idea or it may be of great potential in clinical use.

In spite of the potential use of protozoan-based antitumor therapy, it is important to mention the occurrence of the side effects in the clinical use of live parasites. For example, the infection of *T. gondii* [97], *T. cruzi* [98], or *Plasmodium* [99] may trigger cachexia which remains a devastating problem for cancer patients. As a complex manifestation of systemic inflammation, gut barrier dysfunction, and muscle wasting, cancer cachexia affects the survival and quality of life of patients [100]. One possible solution is to use attenuated pathogens through gene modification, such as *T. gondii* so that the pathogens would be cleared before the induction of other side effects when the host developed acquired immunity (around 1–2 weeks). However, the short duration of infection by the attenuated pathogen may be unable to induce sustainable activation of the immune system. Thus, several doses of attenuated parasites are necessary to achieve anticancer efficacy in clinical use. Another solution is to give a single low dose of a drug, such as artesunate for *Plasmodium* [41], to control the parasite density to a safe level. This may raise the concerns of the drug resistance of artesunate. According to their reports [41], up to now there are no signs of drug resistance in the treatment of over 100 cancer patients, therefore the clinical safety of *Plasmodium* immunotherapy is guaranteed.

**Table 1:** Protozoan parasites with antitumor effect.

Parasite	Cancer	Mechanism of Action	Reference
*Leishmania* spp.	breast cancer; HPV-associated tumors	Activation of CD4+ and CD8+ T cells, macrophages in spleen or NK cell; Induction of proinflammatory cytokines that help the generation of protective Th1 responses; Increasing the levels of IgG2a	[6,81]
*Neospora caninum*	murine thymoma EG7	Activation of NK cell- and CD8-T cell-dependent protective antitumor response; IFN-γ secretion in tumor microenvironment	[7]
*Eimeria* spp.	Sarcoma tumor S180	EA upregulates inflammatory modulators MCP-1, IL-6, IL-12, IFN-γ, and TNF-α	[26]
*Toxoplasma gondii*	Melanoma; Lewis lung carcinoma; Ehrlich’s adenocarcinoma; Pancreatic ductal adenocarcinoma; ovarian carcinoma	Secreted proteins activate antitumor immune responses involving CD4+ and CD8+ T cells, IL-12, IFN-γ and TNF-α or activation of NK cell; Increase the levels of IgG1 and IgG2a; Suppressed the levels of angiogenic factors (VEGF, integrin, MMP2, and MMP9)	[8,12,23,60,95]
*Trypanosoma cruzi*	mammary cancer; colon cancer; Melanoma; Ehrlich’s adenocarcinoma	Calreticulin inhibits vascular endothelial growth factor (VEGF)-induced cell proliferation and induces cell apoptosis; activation of CD4+ and CD8+ T cells and macrophages and DC	[30,101,102,103]
*Plasmodium* spp.	non-Hodgkin’s lymphoma (Karpas299) and prostate cancer (PC-3); Lewis lung cancer; hepatocellular carcinoma; breast cancer	rVAR2 binds with the distinct oncofetal chondroitin sulfate that makes rVAR2 a potential ideal carrier for anti-cancer drug delivery; Activation of NK cell, DC, CD8+ T cell; Suppressed the levels of angiogenic factors (VEGF, MMP9, IGF); Reduce the numbers of MDSC and Treg through CCL17/22-CCR4 pathway	[18,104,105,106]

Above all, the use of genetically modified protozoan parasites and derived molecules combined with chemotherapy could be an important element for promoting antitumor treatment.

## Data Availability

Not applicable.
